# Editorial: Role of transcription factors and sigma factors in bacterial stress physiology

**DOI:** 10.3389/fmicb.2023.1291172

**Published:** 2023-10-06

**Authors:** Ernesto Perez-Rueda, Herb E. Schellhorn, Santosh Kumar

**Affiliations:** ^1^Instituto de Investigaciones en Matemáticas Aplicadas y en Sistemas, Universidad Nacional Autónoma de México, Unidad Académica del Estado de Yucatán, Mérida, Mexico; ^2^Department of Biology, McMaster University, Hamilton, ON, Canada; ^3^Metabolic Engineering and Fermentation Science Group, Department of Food Science, University of Wisconsin-Madison, Madison, WI, United States

**Keywords:** microbial physiology, environmental stress response, gene expression, alternative sigma factors, transcription factors (TFs)

Microorganisms are equipped with genetic information in their DNA essential for duplication to survival in the environment. Transcription is the first committed step in gene expression where DNA is copied into RNA which is then translated into proteins to control various biological processes (Mejía-Almonte et al., 2020). To control the flow of this genetic information, microbial genomes encode a wide diversity of transcriptional regulators that interact and compete with core RNA polymerase to transcribe gene expression to cope with changing environmental conditions (Seshasayee et al., 2011; Browning et al., 2019). The number of these regulators can vary from one microorganism to another depending on their lifestyle and fluctuations in external and internal environmental factors, such as temperature pH, salinity, nutrient supply, and antibiosis. Under ambient conditions, most genes required for metabolic process are controlled by the housekeeping sigma factor, RpoD or σ^70^. However, microorganisms also employ alternative sigma factors such as RpoN or σ^54^ for nitrogen metabolism, RpoH or σ^32^ for heat stress, RpoS for stationary phase stress, and RpoE or σ^24^ to regulate responses to oxidative stress, and other extra-cytoplasmic stresses (Paget and Helmann, 2003). Transcription factors (TFs) belonging to diverse families, such as MarR family, GntR family, TetR family, and CRP/FNR family control expression of antibiotics resistance, pathogenicity, biofilm, and other numerous biological processes (http://web.pcyt.unam.mx/EntrafDB/). These sigma factors and transcription factors differ from each other due to their distinct protein domains involved in interacting with specific binding sites on DNA, called promoters and transcription factor binding sites (repressors and activators sites), respectively (Perez-Rueda et al., 2018). Multiple interacting partners and cross-talk among different regulators can interplay in sensing and controlling the transcription of genes under changing environmental conditions (Rai et al., 2018; Taylor et al., 2022). Therefore, molecular analysis of the role and regulation of transcriptional regulators is central to understand microbial process like stress adaptation, drug resistance, virulence, and disease progression, among others (Schellhorn, 2014; Roncarati et al., 2022).

This Research Topic focuses on the role and regulation of sigma factors and transcription factors governing initiation of gene expression under various physiological and stress conditions. For instance, Lovelace et al. compared the capacity of two common *Salmonella enterica* strains, 14028s and LT2 (strain DM10000) to opportunistically colonize the leaf apoplast of two model plant hosts *Arabidopsis thaliana* and *Nicotiana benthamiana* during disease. In this regard, the authors identified *rpoS* (sigma S)-dependent alterations in the utilization of L-malic acid, an abundant carbon source in *N. benthamiana* apoplastic wash fluid. In addition, data were found to be consistent with higher relative basal values of reactive oxygen species (ROS) in *N. benthamiana* leaves than in *A. thaliana* leaves. Finally, the study indicates that the conducive environment generated by pathogen modulation of the apoplast niche can vary from hosts to host even with a common disease-compatible pathogen.

Bensig et al. identified the gene *srlA* (stress resistance locus A) required for growth on solid media with increased NaCl concentrations in the nitrogen-fixing facultative endosymbiont *Sinorhizobium* (Ensifer) *meliloti*. The encoded protein carries a predicted thioredoxin fold and deletion of the gene also results in increased sensitivity to hydrogen peroxide and cumene hydroperoxide. A deletion mutant yields phenotypic revertants on high salt medium and genome sequencing revealed that all revertants carry a mutation in genes homologous to either *cenK* or *cenR*. *srlA* promoter activity is abolished in these revertant host backgrounds and in a strain carrying a deletion in *cenK*. The authors also observed that the *srlA* promoter is autoregulated, displaying low activity in a wildtype (wt) host background and high activity in the *srl* deletion mutant background. The *srlA* promoter includes a conserved inverted repeat directly upstream of the predicted −35 subsequence. Finally, these results document the first identified CenK–CenR regulon member in *S. meliloti* and demonstrate this two-component regulatory system and gene *srlA* influences cellular growth and persistence under certain stress-inducing conditions.

Costa et al. reconstructed a Gene regulatory network (GRN) in the pathogen *Staphylococcus aureus*. They considered literature-based and comparative genomics approaches to reconstruct the GRN of the high biofilm-producing strain Bmb9393, belonging to one of the highly disseminating successful clones, the Brazilian epidemic clone. In addition, the authors analyzed transcriptomes available in the literature to construct a set of genes differentially expressed in the biofilm, covering different stages of the biofilms and genetic backgrounds of the strains. In total, the GRN comprises 1,803 regulatory interactions between 64 transcription factors and the non-redundant set of 1,151 target genes with the inclusion of 19 new regulons compared to the *S. aureus* strain N315 transcriptional regulatory network published in 2011. Finally, the mapping of the set of genes with altered expression in the biofilm in the Bmb9393 gene regulatory network would help to depict how different growth modes can alter the regulatory systems. The data revealed 45 transcription factors and 876 shared target genes. Thus, the gene regulatory network model provided represents the most up-to-date model for *S. aureus*, and the set of genes altered in the biofilm provides a global view of their influence on biofilm formation from distinct experimental perspectives and different strain backgrounds.

Finally, Hołówka et al. analyzed the Nucleoid-associated proteins (NAPs) associated to the organization of bacterial chromatin and regulating gene expression. In particular, the authors used super-resolution microscopy, to perform a comprehensive analysis of the roles of HupB and mIHF in chromosome organization in *Mycobacterium smegmatis*. They described that HupB is a structural agent that maintains chromosome integrity on a local scale, and that the lack of this protein alters chromosome morphology. In contrast, mIHF is a highly dynamic protein that binds DNA only transiently, exhibits susceptibility to the chromosomal DNA topology changes and whose depletion leads to the growth arrest of tubercle bacilli. Additionally, the depletion of *Mycobacterium smegmatis* integration host factor (msIHF) leads to chromosome shrinkage and replication inhibition.

## Author contributions

EP-R: Writing—original draft, Writing—review and editing. SK: Writing—original draft, Writing—review and editing. HS: Writing—original draft, Writing—review and editing.
